# High Nanodiamond Content-PCL Composite for Tissue Engineering Scaffolds

**DOI:** 10.3390/nano10050948

**Published:** 2020-05-15

**Authors:** Kate Fox, Rahul Ratwatte, Marsilea A. Booth, Hoai My Tran, Phong A. Tran

**Affiliations:** 1 Center for Additive Manufacturing, School of Engineering, RMIT University, Melbourne, VIC 3000, Australia; rahul.ratwatte@unimelb.edu.au (R.R.); marsilea.harrison@rmit.edu.au (M.A.B.); 2 Interface science and materials engineering group, School of Mechanical, Medical and Process Engineering, Queensland University of Technology (QUT), 2 George Street, Brisbane QLD 4000, Australia; hoaimy.tran@qut.edu.au; 3 Institute of Health and Biomedical Innovation, Queensland University of Technology, Kelvin Grove, QLD 4059, Australia

**Keywords:** nanodiamond, polycaprolactone, composite, 3D-printed scaffold

## Abstract

Multifunctional scaffolds are becoming increasingly important in the field of tissue engineering. In this research, a composite material is developed using polycaprolactone (PCL) and detonation nanodiamond (ND) to take advantage of the unique properties of ND and the biodegradability of PCL polymer. Different ND loading concentrations are investigated, and the physicochemical properties of the composites are characterized. ND-PCL composite films show a higher surface roughness and hydrophilicity than PCL alone, with a slight decrease in tensile strength and a significant increase in degradation. Higher loading of ND also shows a higher osteoblast adhesion than the PCL alone sample. Finally, we show that the ND-PCL composites are successfully extruded to create a 3D scaffold demonstrating their potential as a composite material for tissue regeneration.

## 1. Introduction

There is a growing need for effective scaffolds for tissue regeneration in biomaterials research, with requirements including biocompatibility, strength, and structure. Detonation nanodiamonds (NDs) are gaining significant interest for their mechanical strength, optical properties, and biocompatibility [1,2,3,4]. NDs have been assessed for biocompatibility both in vitro and in vivo with positive outcomes such as low cytotoxicity [5,6,7], improved cellular adhesion [8], and improved cell proliferation [3]. They are significantly less cytotoxic than other carbon-based nanoparticles such as CNTs [7], and endocytic NDs are non-cytotoxic during cell division and differentiation [9]. Further to their biocompatibility, NDs exhibit other beneficial properties. Fluorescence in ND particles allows for non-invasive fluorescence tracking in both cells [10] and tissue [4]. The large specific surface area and unique surface structure of NDs are used to make drug delivery systems [11,12,13]. Antibacterial properties are also displayed by NDs, mainly due to surface chemical terminations [14,15,16,17]. Furthermore, NDs have been shown to prevent biofilm formation, particularly when combined with carbohydrates [18,19]. The low toxicity, improved cellular interactions, and biocompatibility make them an attractive material for composite tissue engineering scaffolds.

The structure of NDs consists of a diamond-like core with a graphitic outer shell that contains many oxygen-rich functional groups, attractive for developing polymer composites [20]. The addition of NDs into a polymeric matrix has been shown to enhance mechanical properties [21], including hardness and elastic modulus [22], abrasion and scratch resistance in an epoxy polymer matrix [23], and compressive strength in a poly(vinyl alcohol) matrix [22]. Polycaprolactone (PCL) is an FDA-approved polyester widely studied for soft and hard tissue engineering scaffolds because of its non-toxic nature, degradability, and low melting temperature [24]. Despite those excellent properties, this polymer still possesses limitations, including a slow degradation time (2–3 years in interstitial fluid [25]), low mechanical strength, and high hydrophobicity [26,27], which hinder cell interactions [3,4,25]. Co-polymers and blended polymer composites are shown to improve PCL properties, particularly to tailor degradation properties [28]. A composite of PCL, poly(lactide-*co*-glycolide) (PLGA) and tricalcium phosphate showed a faster degradation speed [29], while a co-polymer of PCL and δ-valerolacton also exhibited faster degradation rates than PCL alone [30]. A review by Bartnikowski et al. covers different PCL degradation mechanisms within physiological contexts in detail [28]. Modification of PCL, as an ideal biomaterial for scaffold design, thus involves reduction of its hydrophobicity and modification of degradation rate to be in harmony with tissue regeneration rate [25].

In this study, we use NDs in a PCL matrix in order to make a biodegradable composite (ND-PCL) with improved cellular interactions. This builds upon the previous work within our group in which we formed a 0.1% wt ND-PCL composite [4]. Here we extend the ND loading to 10% wt and 20% wt and determine that ND incorporation affects the physicochemical properties of PCL, namely the mechanical and biodegradation properties of the ND-PCL composite. Lastly, we use osteoblasts to investigate the cellular response to the composite. We chose osteoblasts as a relevant cell type since the composite scaffolds we form herein are aimed for bone regeneration e.g., maxillofacial surgical implants. This builds upon more traditional ND-PCL film fabrication methods such as evaporation giving free-standing thin films [4] and electrospinning [3,21,31]. Herein we instead use additive manufacturing thermal extrusion-based 3D printing to show spatial control of the ND-PCL composites. Additive manufacturing techniques allow for design tailoring [25,32], scaffold porosity, and personalized patient care [33]. We show that ND-PCL composites have great potential as scaffolds for tissue engineering, displaying good biocompatibility, degradation properties and processability.

## 2. Materials and Methods

### 2.1. Fabrication of ND-PCL Composites

**Fabrication of ND-PCL composites.** The PCL (Mn = 80,000) was from Sigma Aldrich (Castle Hill, NSW, Australia); NDs of 45 nm were obtained from NaBond (Nabond Technologies, China) and irradiated as detailed in [34]. In brief, as-received ND were dispersed in deionized water at a concentration of 1 mg mL^−1^. Centrifugation was used to remove large aggregates prior to irradiation with high energy electrons (2 MeV to a total fluence of 1 × 10^18^ cm^−2^). The ND material was then annealed in a vacuum at 800 °C for 2 h in order to induce vacancy diffusion and ND formation. ND-PCL composites were produced as films for characterization, biological compatibility testing and additive manufacturing. The fabrication process involved physical blending of ND and PCL at 5%, 10% and 20% ND wt% in trichloromethane. The mixture suspension was cast onto glass dishes and free-standing composite films (~0.1 mm) were removed after complete solvent evaporation.

### 2.2. Characterization of ND-PCL Composites

**Physicochemical characterization.** Scanning electron microscope images were used to determine sample morphology. Samples were studied under the scanning electron microscope (FET Quanta ESEM (Thermo Fisher, OR, USA) 30 kV accelerating voltage, with a working distance of 10.6 mm, spot size 5 in variable (VP) pressure mode). Static water contact angle measurements were used [35] for 3 samples per condition and 3 repeats per sample, with the results then averaged. Samples (5 mm Ø) were pre-treated via immersion in ethanol 80% for 2 h and left to evaporate until completely dry, and mounted onto glass slides for examination [36]. Fourier-transformed infrared spectroscopy analysis was performed on a Nicolet FTIR spectrophotometer (Nicolet Analytical Instruments (Thermo Fisher, OR, USA)) using a setting of 64 scan-average and a resolution of 1 cm^−1^. Thermal analysis differential scanning calorimetry (TA Instrument, DSC Q100 (Rydalmere NSW, Australia)) was used to measure the melting temperature (Tm) in order to evaluate the crystallinity of the polymer. Pure PCL and treated PCL samples (4–5 mg) were heated at rate of 10 °C/min from −60 °C to 100 °C, and the crystallinity calculated based on the equation [37,38]:

(1) $$\chi \left(\%\right)=\left(\frac{∆H_{sample}}{∆H_{0}}\right)\times 100$$

where: $\chi \left(\%\right)\;$ is the crystallinity percentage, $∆H_{sample}$ is the sample’s enthalpy of fusion and $∆H_{0}\;$ is the enthalpy of fusion for a 100% crystalline PLC, with a value of 136.1 J/g.

**Tensile strength measurement.** The mechanical properties of the films were examined using an Instron 4302 Material Testing System operated by Series IX Automated Materials Tester version 7.43 system software with a 1 kN load-cell. Samples were cut into dog bone shapes (12.7 mm width, 38 mm length in the middle section, 79 mm total length) in accordance with the ASTM D695-96 guidelines and subject to elongation at a rate of 1 mm/min until failure.

**Accelerated in vitro degradation.** Sodium hydroxide (2 M) was used to accelerate the hydrolysis reaction [39]. Replicates of 14.5 to 16.0 mg pieces of PCL and ND-PCL films (24) were submerged in 2.0 mL NaOH in closed Eppendorf tubes, and maintained at 37 °C. At time points (12, 24, 48 and 72 h) the films were removed and rinsed thoroughly with de-ionized water. The samples were then dried, placed in an oven at 35 °C for 48 h and weighed to calculate the percentage mass loss.

### 2.3. Investigating Cellular Interactions with ND-PCL Composites and Performing Additive Manufacturing

**Cell culture.** Cell culture was performed using primary human osteoblasts (between 2–6 passages) cultured in DMEM supplemented with 10% fetal bovine serum and 1% penicillin/streptomycin. For the adhesion assay, cells were seeded onto 5 mm Ø film samples at a density of 800 cells/sample and incubated for 4 h. Cells were fixed in a 4% formaldehyde, permeabilised with 0.2% Triton X-100/PBS, stained with 0.5% BSA/PBS containing 0.8 ug/mL TRITC-conjugated phalloidin and 5 µg/mL DAPI, and imaged.

**Additive Manufacturing.** The 20 wt% ND-PCL composite was used to make 3D scaffolds using a layer-by-layer melt-screw extrusion through a 20-gauge needle at an extrusion temperature of 175 °C. The composites are printed using a custom-made bioextruder.

### 2.4. Statistical Analysis

The results are reported as mean and standard deviations and a student *t*-test is used to analyze statistical significance between means.

## 3. Results

### 3.1. Physicochemical Properties and Characterization of ND-PCL Composites

**Figure 1:**
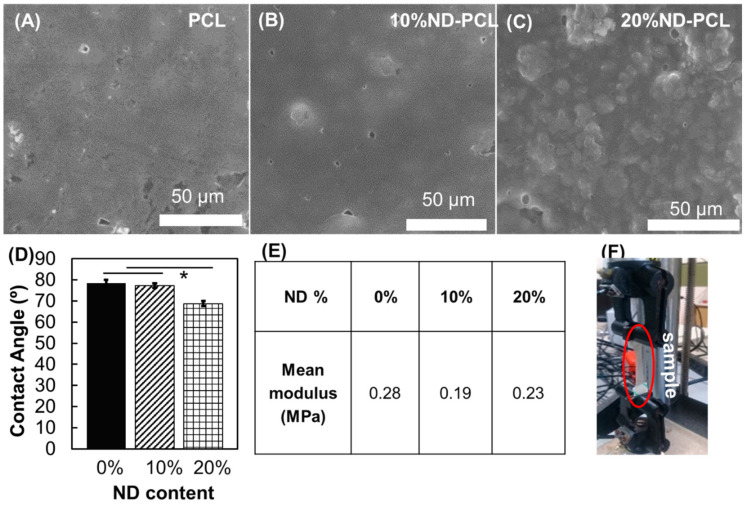
(**A**–**C**) SEM surface imaging of (**A**) polycaprolactone (PCL) (**B**) nanodiamond-polycaprolactone (ND-PCL) films with 10% ND loading and (**C**) 20% ND loading. (**D**) Contact angle measurement (n = 3, student *t*-test (*p* ≤ 0.05)). (**E**) Mean tensile modulus for films with different ND loadings (n ≥ 6) and (**F**) setup of the tensile test.

Figure 1A–C shows the surface morphology at different ND loading conditions as investigated by scanning electron microscopy. PCL samples showed a smooth surface with large and uneven domains, whilst the addition of NDs resulted in a change in surface topology. The addition of 10% ND to PCL resulted in surface particle formation, most likely ND clusters. Meanwhile, at 20% ND loading a rougher surface with several granular clusters could be seen. The addition of 20% ND to the PCL showed a significant (*p* ≤ 0.05) increase in the wettability of the films, while the 10% ND-PCL composite film did not show a large difference from PCL film wettability (Figure 1D). The material tensile properties decreased as the ND *w*/*w* fraction increased (Figure 1E,F). The elastic modulus of both composite conditions produced lower stiffness compared to PCL, with the 20% *w*/*w* ND-PCL composite closest to that of PCL.

**Figure 2:**
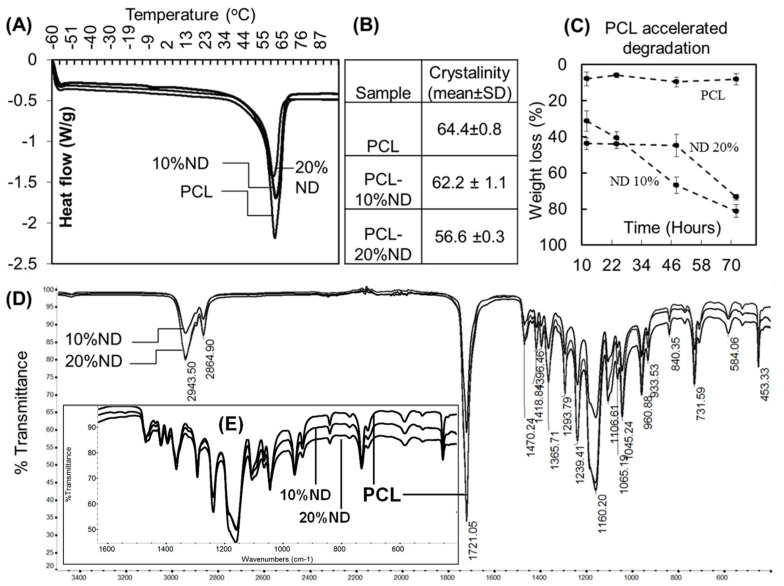
(**A**,**B**) Crystallinity of PCL and ND-PCL composites and their representative differential scanning calorimetry (DSC) traces (n = 5). (**C**) Weight loss of samples in accelerated degradation experiments (n = 6). (**D**) Representative FTIR spectra of PCL and ND-PCL composites. (**E**) Inset showing FTIR spectra in the 400–1600 cm^−1^.

Differential scanning calorimetry showed that the crystallinity of PCL decreased as the ND content increased. PCL had a percentage crystallinity of 64% which reduced to 62% for 10% ND-PCL and to 57% for 20% ND-PCL (Figure 2A,B). However, both ND-PCL composite samples showed significant degradation over the observed time period (Figure 2C). The 20% ND-PCL composite showed an initial loss of ~40% mass, followed by a slowed loss before a final mass loss of 74% after 70 h. FTIR spectra showed no significant difference in chemical bonding, indicating that the interaction between ND and PCL uses physical bonds (Figure 2D).

### 3.2. Cellular Interactions with ND-PCL Composites and Additive Manufacturing of Composites

**Figure 3:**
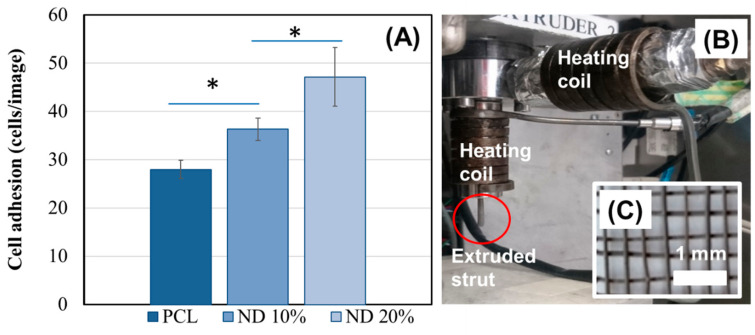
(**A**) Osteoblast adhesion on composite materials showing higher adhesion compared to PCL alone (mean ± S.E.M, n = 6, student *t*-test (*p* ≤ 0.05)). (**B**) Proof of concept of the 3D printing compatibility of PCL-ND 20% composite showing the melt-extrusion setup and (**C**) a scaffold printed using layer-by-layer deposition of extruded struts (nozzle size: 20-gauge needle).

A cell adhesion assay was used to determine the surface interactions between human osteoblasts and the composite ND-PCL samples. Figure 3A shows a marked improvement in the attachment of human osteoblast cells after the 10% and 20 wt% ND composites are added to the PCL films (Figure 3A). The cells also appeared to remain viable on the composite materials after 14 days in culture (Figure S1). A proof of concept printing trial was performed to investigate whether including ND into PCL enables extrusion and additive manufacturing of ND-PCL composites. Figure 3B shows the results of an extruded 20% ND loaded composite film. The extruded ND-PCL composite appeared to hold its shape with no limitation in flow of the composite through the extrusion nozzle (Figure 3B).

## 4. Discussion

### 4.1. Inclusion of ND into a PCL Scaffold Modifies the Material Surface

As the interest in tissue engineering increases new materials are required to improve the interface. Here we show a scaffold composed of polycaprolactone and detonation nanodiamonds, ND-PCL, with ND inclusion of both 10% ND-PCL composite and 20% ND-PCL. By adding ND into the PCL, the newly formed composite material reports a lower tensile strength and decrease in crystallinity coupled with faster degradation when we compare the two composites to the PCL alone. Further, the degradation profiles of the 10% and 20% ND-PCL composites are different with the 20% ND composite able to resist dissolution for a longer time period than the 10% ND composite. This is because NDs are likely to supply additional surface nucleation sites during film drying, increasing the surface roughness and hence the hydrophilicity of the material. As expected there is a limited mass decrease that occurs for PCL during the 70 h exposure to NaOH [40]. Increased and tunable degradation profiles are useful for implantable biomaterials, where material degradation occurs ideally in a controlled manner. The chosen biomaterial should degrade at a rate aligned with the rate of tissue regeneration [25]. Our results show that a degradation rate can be tailored by using composite ND-loading to meet requirements. Our previous results show ND-PCL composites exhibit the potential for tracking degradation in situ via sub-dermal fluorescent imaging [4]. The ability to track and even tune a timely degradation of material is highly coveted for biomedical implant scaffolds.

### 4.2. Inclusion of ND into a PCL Scaffold Modifies Biointerface

As ND is incorporated into the PCL material, the biointerface appears to be more supportive of osteoblast adhesion, with a clear improvement in cell attachment with increased ND loads. Osteoblasts are an established key in the bone regeneration cycle and as such it is important that the new composite material can support their attachment and proliferation. The improvement in adhesion with 20% ND compared to the PCL is likely linked to the improved hydrophilicity of the ND-PCL scaffold. Hydrophilicity is an important property for scaffold biomaterials. This result supports our previous work [4] where ND-PCL was found to be a superior scaffold for fibroblast attachment, and that of others [3,25] who observe that improvements in PCL-composite hydrophilicity by adding ND can equate to better cellular adhesion. This is also the case with osteoblasts as well as fibroblasts [4,25] and Chinese hamster ovarian (CHO) cells [3]. The biointerface shows an increased surface roughness as ND content increases, contributing to the increased hydrophilicity. This supports the finding of Jeon et al. [41], who used oxygen plasma treatment on PCL scaffolds to tailor a range of surface roughness topologies, that the surface roughness enhanced initial cell adhesion [41]. The increase in roughness of our ND-PCL composites as compared to PCL is a likely contributor to the improved osteoblast cell adhesion observed and a vital first step towards promoting osseointegration.

### 4.3. Fabrication and Additive Manufacturing of ND-PCL Scaffolds

The fabrication of ND-PCL composites is limited by the fabrication techniques used to manufacture the composites. Using casting and electrospinning, significantly lower ND concentrations of 0.1–6% ND *w*/*v* [3,4,21,42] are used to make composites, while herein we are increasing this to 20% ND *w*/*w* loading. Here, we use the ND-PCL composite as a base material in a custom-made bioextruder for additive manufacturing. Following optimization, it was found that the 20% ND-PCL blend can be effectively extruded through a 20-gauge needle (internal diameter of 603 µm [43]), providing the ability to fabricate customized scaffold designs. A simple design was used as a prototype scaffold (Figure 3C); however, the 3D printing of scaffold design has powerful capabilities. This increase in ND wt% produces beneficial changes in the physical properties of the scaffold; its degradation profile and its hydrophilicity, thereby improving the biointerfacial cellular interactions. Since the aim is to use these scaffolds for tissue engineering, the higher ND content should also be considered in terms of toxicity and clearance. Although a higher concentration of ND is used, the size of a scaffold is likely to be small. However it is important to note that NDs are shown to have high biocompatibility and low toxicity [5,6,7,9]. However, clearance is an important issue to consider. Fortunately, the inherent fluorescence in NDs can be used to investigate in situ degradation of the scaffold as highlighted by our previous work [4], and in vivo imaging [10]. Composites loaded with other diamond structures [44], or other base polymers [31] have incorporated higher material loadings, with other applications in mind and without the beneficial properties of NDs or PCL.

When designing biomedical tissue engineering scaffolds, it is best to control morphology at multiple structural levels to meet clinical requirements [25]. This can be thought of in two scales, macroscale and microscale. Macroscale can include the external architecture of the implant, mechanical properties, and scaffold density. Microscale, on the other hand, can refer to the material porosity, surface topology, and the degradation capabilities of the material. Changes to processing parameters can be used in additive manufacturing to tailor PCL scaffolds [25,32,39]. Additive manufacturing can contribute to control of the microscale via porosity of the printed scaffold. Large-scale porosity can be adjusted depending on printing conditions and design. A balance needs to be achieved to maintain advantageous mechanical properties while promoting osteoblast migration, integration, nutrient transfer, and vascularization. Regeneration occurs as cells grow either within the scaffold itself or shifting from neighboring tissue, highlighting the importance of the scaffold microstructure. On the macroscale, additive manufacturing can greatly improve the ease of design, precision, resolution, and individualization of biomedical implants. 3D printing coupled to 3D scanning can potentially offer solutions for patient-specific care [33], with implants able to match shape requirements and, therefore, improve implant success.

Our future work based on this current proof-of-principle study will focus on investigating more complex designs for 3D printing, and subsequently test these complex designs for their physicochemical properties and cellular interactions. Characterization of the 3D printed structures must consider printed structure, surface roughness, contact angle measurement, mechanical characterization, and degradation studies. Mechanical properties and mass loss are strongly influenced by construct geometry; therefore the degradation rate of 3D structures may show interesting behavior [28]. Coatings and additions to improve scaffold use for bone regeneration may also be considered [45,46]. Subsequent steps include cellular interactions of ND-PCL 3D printed composites with osteoblasts and finally, in vivo testing of promising composites. The results from this study highlight the exciting potential of ND-PCL composites. By combining fluorescent degradation tracking, tunable degradation profile, improved surface wettability, and additive manufacturing capacity, ND-PCL composites have high potential for tissue regeneration scaffolds.

## 5. Conclusions

Here, we investigated the potential of ND-PCL composites as a biomaterial for tissue engineering scaffolds. Our findings showed that high loading of ND in PCL was possible (up to 20% *w*/*w*), and this changed the physicochemical properties of the composite. Tensile properties decreased slightly, while a marked increase in degradation was observed after ND incorporation. The hydrophilicity of the composite was greatly increased after ND addition, likely a feature of surface roughness. This, in turn, contributes to the increased adhesion of osteoblast cells observed on ND-PCL composites as compared to PCL. Lastly, ND-PCL composite could be additively manufactured into 3D scaffolds via melt-extrusion, paving the way for preparation of advanced tissue engineering scaffolds.

## Supplementary Materials

The following are available online at https://www.mdpi.com/2079-4991/10/5/948/s1, Figure S1: Representative images of cells cultured on PCL, ND-PCL 10% and ND-PCL 20% for 2 weeks.
