# Supplementary material for: High Nanodiamond Content-PCL Composite for Tissue Engineering Scaffolds
Source: Nanomaterials (Basel). 2020 May 15;10(5):948. doi: 10.3390/nano10050948 (PMC7279315; doi:10.3390/nano10050948)
Supplement: Supplementary file 1 [file nanomaterials-10-00948-s001.pdf]

nanomaterials

Article

High Nanodiamond Content-PCL Composite for Tissue Engineering Scaffolds

Supplementary Materials

Kate Fox [1,*], Rahul Ratwatte [1], Marsilea A. Booth [1], Hoai My Tran [2,3] and Phong A. Tran [2,3,*]

1 Center for Additive Manufacturing, School of Engineering, RMIT University VIC 3000, Australia; rahul.ratwatte@unimelb.edu.au (R.R.); marsilea.harrison@rmit.edu.au (M.A.B.)
2 Interface science and materials engineering group, School of Mechanical, Medical and Process Engineering, Queensland University of Technology (QUT), 2 George Street, Brisbane QLD 4000, Australia; hoaimy.tran@qut.edu.au
3 Institute of Health and Biomedical Innovation, Queensland University of Technology, Kelvin Grove QLD 4059, Australia
* Correspondence: kate.fox@rmit.edu.au (K.F.); phong.tran@qut.edu.au (P.A.T.)

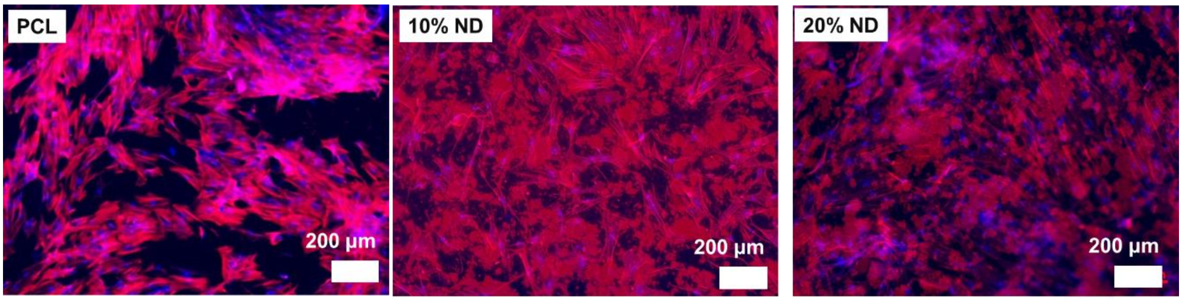

Figure S1. Representative images of cells cultured on PCL, ND-PCL 10% and ND-PCL 20% for 2 weeks. DAPI (blue) and rhodamine phalloidin (red) were used to stain cell nuclei and F-actin in cell cytoskeleton, respectively. Some background red particle fluorescence is observed in the 10% and 20% ND samples.
